# Effects of the Supplementation of *N*-acetylcysteine on Redox Imbalance, Cytokine Levels, and Perinatal Outcomes in Pregnant Women with Preeclampsia: A Double-Blind, Randomized, Placebo-Controlled Pilot Study

**DOI:** 10.3390/ph19091344

**Published:** 2026-08-25

**Authors:** Danielle Alice Vieira da Silva, Micaely Cristina dos Santos Tenório, Fabiana Andréa Moura, Nassib Bezerra Bueno, Nathálya da Silva Severino, Alexandra Rodrigues Bezerra, Bianca Gomes de Souza, Marilene Brandão Tenório Fragoso, Tauane Alves Dutra, Orlando Roberto Pimentel de Araújo, Amylly Sanuelly da Paz Martins, Marília Oliveira Fonseca Goulart, Alane Cabral Menezes de Oliveira

**Affiliations:** 1 Institute of Biological and Health Sciences, Federal University of Alagoas, Maceió 57072-970, Brazil; danielle.silva@fanut.ufal.br (D.A.V.d.S.); alexandra_rbezerra@hotmail.com (A.R.B.); tdutra7@gmail.com (T.A.D.); 2 Institute of Chemistry and Biotechnology, Federal University of Alagoas, Maceió 57072-970, Brazil; micaely.tenorio@hotmail.com (M.C.d.S.T.); marilene.tenorio@iqb.ufal.br (M.B.T.F.); orlando_rpa@hotmail.com (O.R.P.d.A.); mariliaofg@gmail.com (M.O.F.G.); 3 Northeast Biotechnology Network (RENORBIO), Federal University of Alagoas, Maceió 57072-970, Brazil; amyllymartins@gmail.com; 4 College of Nutrition, Federal University of Alagoas, Maceió 57072-970, Brazil; nassibbb@hotmail.com (N.B.B.); nathalya.severino@fanut.ufal.br (N.d.S.S.); biagomes191@gmail.com (B.G.d.S.)

**Keywords:** *N*-acetylcysteine, pregnancy, antioxidant, anti-inflammatory

## Abstract

**Background/Objectives**: This study aimed to evaluate the effects of oral administration of *N*-acetylcysteine (NAC) in pregnant women with preeclampsia (PE), using serum markers of redox imbalance and cytokine levels and perinatal outcomes. **Methods**: A double-blind, randomized, placebo-controlled pilot study was carried out from 2021 to 2023. The study was retrospectively registered with REBEC (Brazilian Clinical Trials Registry). After screening, women were randomized into 2 groups according to the type of supplementation: 600 mg of NAC (NAC group) or capsules containing 2% colloidal silicon dioxide and 98% microcrystalline cellulose (placebo group) until delivery. Blood samples were collected to quantify biomarkers of redox imbalance and cytokine levels. Questionnaires containing socioeconomic, clinical, obstetric, and anthropometric data were applied. **Results**: A total of 51 pregnant women with PE were included (26 in the placebo group and 25 in the NAC group). The use of NAC was associated with a higher gestational age at birth (GA) of the conceptus (placebo group: 37.39 weeks ± 1.96 SD vs. NAC group: 38.45 weeks ± 1.51 SD; *p* = 0.044). A moderate effect size was observed for this variable (Cohen’s *d* = 0.52). However, treatment allocation (NAC vs. control) was not independently associated with gestational age at birth after adjustment for covariates (F(1,28) = 0.144; *p* = 0.707; *η*^2^p = 0.005). The adjusted mean difference between groups was 0.92 weeks (95% CI: −0.16 to 2.00). No other significant effects on redox and inflammatory markers were identified using NAC. **Conclusions**: NAC supplementation did not impact the mode of delivery between the NAC and placebo groups. As a secondary exploratory finding, a possible trend toward prolonged gestational age was observed in the NAC group; however, this result should be interpreted with caution, given the exploratory nature of the analysis, the neutrality of the primary outcome, and the limited sample size.

## 1. Introduction

Preeclampsia (PE) is a multifactorial, pregnancy-specific disease responsible for a significant proportion of adverse short- and long-term outcomes for women and their children, such as premature birth, placental abruption, and postpartum hemorrhage [1,2,3]; in addition to maternal death, maternal target organ damage, intrauterine growth restriction (IUGR), respiratory distress syndrome, stillbirths, and neonatal deaths [4]. Affecting approximately 4 to 5% of all pregnancies worldwide [5,6,7,8], this syndrome is characterized by hypertension that develops after the 20th week of gestation. It may be associated with proteinuria, maternal acute kidney injury, hepatic or neurological dysfunction, hemolysis, thrombocytopenia, and IUGR [9].

The pathophysiology of preeclampsia remains incompletely elucidated. However, findings already point to multiple conditions involving redox imbalance, with a greater generation of reactive oxygen and nitrogen species (RONS), endothelial dysfunction, and inflammation, all associated with worse maternal and fetal outcomes [10,11,12].

Given these findings, a therapeutic strategy to minimize damage resulting from PE would be antioxidant intervention, aimed at reducing oxidative stress and, consequently, oxidative damage [11,13,14]. Among these strategies, the use of *N*-acetylcysteine (NAC) stands out for its antioxidant properties, particularly its modulation of reduced glutathione (GSH) levels, and has therefore been tested in several clinical settings, including chronic obstructive pulmonary disease, heart failure, paracetamol overdose, and schizophrenia [15,16,17]. In addition, this compound exhibits anti-inflammatory effects by reducing pro-inflammatory cytokines, thereby reinforcing its therapeutic potential in PE [11,18].

The potential therapeutic role of *N*-acetylcysteine (NAC) in preeclampsia has been explored in clinical trials in the Netherlands and Egypt; however, these studies yielded inconclusive data on its impact on oxidative stress markers and maternal and perinatal outcomes. Therefore, further studies are necessary to clarify its clinical efficacy [19,20,21,22].

Although PE is closely linked to redox imbalance and pro-inflammatory activity, clinical studies involving antioxidant therapy remain scarce and present inconclusive results, highlighting the need to continue these investigations. Thus, this study aimed to evaluate the effects of NAC supplementation on oxidative stress, cytokine levels, and maternal and perinatal outcomes in pregnancies with PE.

## 2. Results

**Figure 1:**
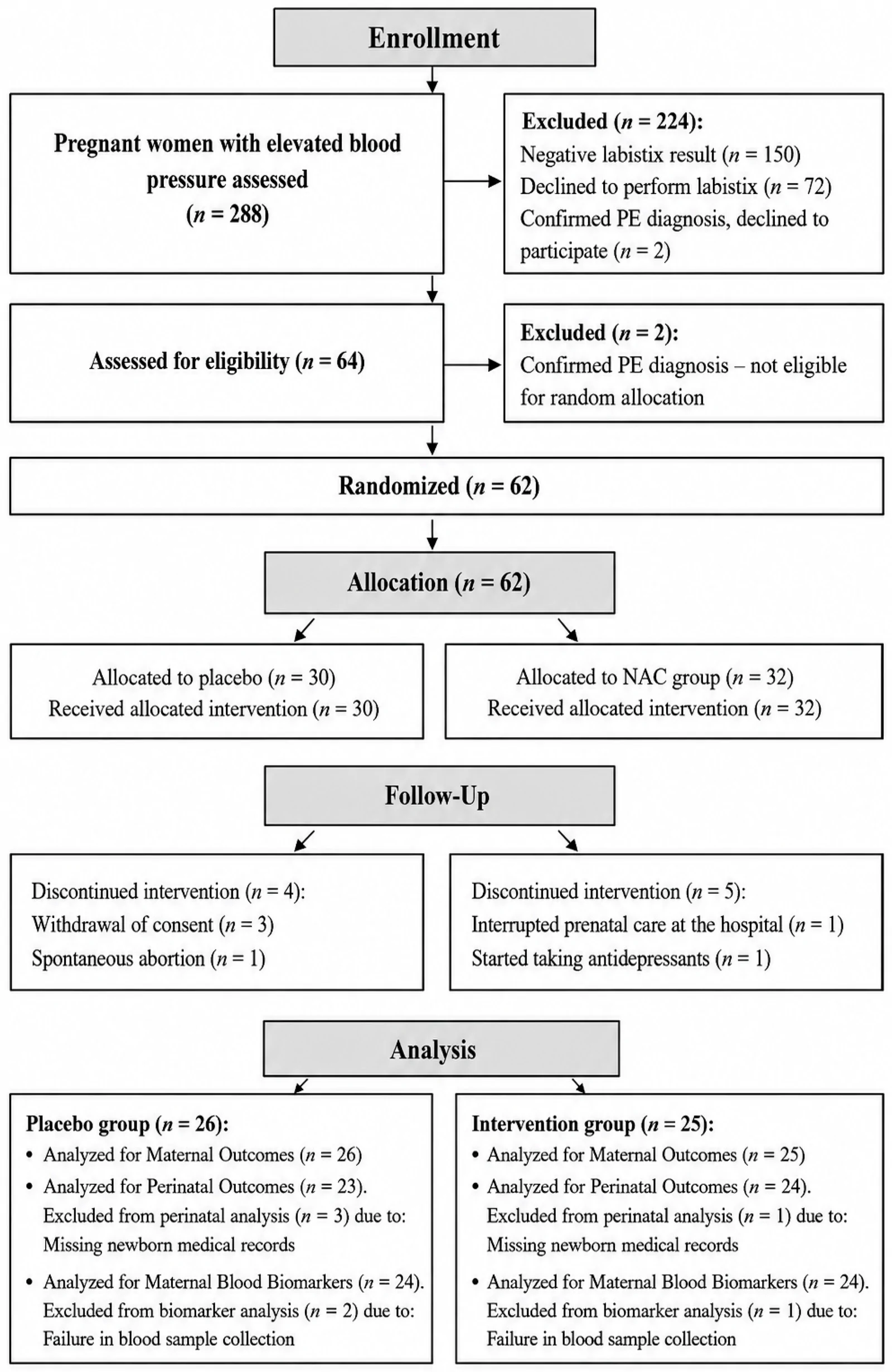
CONSORT flow diagram of participant disposition throughout the study. Pregnant women with elevated blood pressure were assessed (*n* = 288), of whom 224 were excluded due to a negative urine dipstick (labistix) result (*n* = 150), refusal to undergo the urine dipstick test (*n* = 72), or confirmed diagnosis of preeclampsia (PE) with refusal to participate (*n* = 2). Of the 64 women assessed for eligibility, 2 were excluded due to a confirmed diagnosis of PE, resulting in 62 women randomized to the placebo (*n* = 30) or N-acetylcysteine (NAC) (*n* = 32) groups.

The flowchart presents all steps of the trial process (Figure 1). A total of 288 pregnant women with elevated blood pressure were screened, of whom 224 were excluded (150 with a negative urine dipstick result, 72 who declined to perform the test, and 2 with a confirmed PE diagnosis who declined to participate). Of the 64 pregnant women assessed for eligibility, 2 were excluded due to a confirmed diagnosis of PE without eligibility for random allocation, leaving 62 participants randomized (30 to the Placebo group and 32 to the NAC group).

During follow-up, 4 participants discontinued the intervention in the Placebo group (3 withdrew consent and 1 experienced a spontaneous abortion). In the NAC group, 5 participants discontinued the intervention (1 interrupted prenatal care at the hospital and 1 initiated antidepressant therapy).

For the analysis of maternal outcomes, 26 pregnant women were analyzed in the Placebo group and 25 in the NAC group. In the blood biomarker analysis, sample sizes varied according to the specific assay, with data available for up to 24 participants per group (excluding 2 in the Placebo group and 1 in the NAC group due to blood collection failure). For perinatal outcomes, 4 mother–infant dyads were excluded (3 in the Placebo group and 1 in the NAC group) due to delivery occurring in other healthcare facilities, which precluded access to the newborns’ medical records. This resulted in 23 and 24 cases analyzed for perinatal outcomes in the Placebo and NAC groups, respectively (Figure 1).

Regarding socioeconomic and housing conditions, when comparing pregnant women supplemented with NAC and those who used a placebo, the following results were observed, respectively: 64.0% vs. 65.3% (*p* = 0.523) were between 20 and 34 years of age; 54.2% vs. 61.5% (*p* = 0.775) reported a monthly family income equal to or less than one minimum wage; 28.0% vs. 19.2% (*p* = 0.242) had incomplete primary education; and 56.0% vs. 50.0% (*p* = 0.781) were from the state capital. Therefore, the groups did not differ significantly in chronological age, origin, occupation, education, marital status, per capita income, or other variables characterizing the sample (Table 1).

Of the total participants, only two presented with chronic hypertension superimposed on preeclampsia. Regarding medication use, the majority were taking methyldopa. No use of other medications, including antiplatelet agents, was recorded.

The diagnosis of PE was established according to the study protocol, and supplementation with placebo or NAC was initiated immediately after diagnostic confirmation and randomization, with no relevant time interval between diagnosis and initiation of the intervention. The mean duration of supplementation was 76 ± 25.63 days (median 74.0; range 30–128) in the Placebo group and 86.68 ± 30.42 days (median 78.0; range 37–149) in the NAC group, with no statistically significant difference (independent *t*-test: *t* = 1.272; *p* = 0.209), corresponding to the period from inclusion in the trial until the time of delivery. Regarding the primary outcome (mode of delivery), NAC supplementation did not significantly affect it. However, in an exploratory analysis of secondary outcomes, it was observed that NAC supplementation was associated with a significantly higher gestational age (GA) at birth (placebo group: 37.39 weeks ± 1.96 SD vs. NAC group: 38.45 weeks ± 1.51 SD; MD 1.06 weeks; 95% CI: 0.03 to 2.09; *p* = 0.044). A moderate effect size was observed for this variable (Cohen's *d* = 0.52). Overall, 38.2% of deliveries in the study population were preterm, confirming the elevated risk of preterm birth as a consequence of PE (Table 2). Regarding typical maternal clinical conditions of preeclampsia, the groups maintained similar average systolic and diastolic blood pressure levels, with no statistically significant differences between the interventions. In a supplementary ANCOVA, adjusted for gestational age at admission, duration of supplementation, maternal age, BMI, and blood pressure, the mean gestational age at birth was 38.4 weeks (95% CI: 37.6–39.1) in the NAC group and 37.5 weeks (95% CI: 36.7–38.2) in the control group, with an adjusted mean difference of 0.92 weeks (95% CI: −0.16 to 2.00; *p* = 0.093). These data suggest a possible trend toward an association between NAC supplementation and greater prolongation of pregnancy; however, after adjustment, this difference did not reach statistical significance.

Regarding the evaluation of the stress and inflammation biomarkers, no differences were found between the groups for maternal plasma levels before and after supplementation: H_2_O_2_ (*p* = 0.738), SOD (*p* = 0.634), CAT (*p* = 0.593), MDA (*p* = 0.360), GSH (*p* = 0.212), GSSG (*p* = 0.210), GSH/GSSG ratio (*p* = 0.707), MPO (*p* = 0.875), TNF-α (*p* = 0.509), IL-10 (*p* = 0.239), IL-22 (*p* = 0.604) and IL-17 (*p* = 0.348) (Table 3).

When evaluating the impact of NAC on the typical clinical manifestations of PE throughout the trial, taking into account the initial evaluation and all other follow-up visits with the pregnant women, it was found that the Prevalence Ratio (PR) of high blood pressure in the placebo group in relation to the NAC group was 0.901 95% CI [0.66; 1.23], *p* = 0.51; for the headache variable, 0.84 95% CI [0.57; 1.24], *p* = 0.38; for scotomas 0.75 95% CI [0.41; 1.34], *p* = 0.52, and for epigastric pain, 0.52 95% CI [0.27; 1.002], *p* = 0.051 (Table 4).

The prevalence of reported adverse effects attributed to supplementation was low, namely: headache (*n* = 5; *n* = 3 in the placebo group and *n* = 2 in the NAC group); nausea (*n* = 4 in the placebo group); hyperpigmented urine (*n* = 1 in the NAC group); and drowsiness (*n* = 3 in the NAC group). During the trial, there was one recorded miscarriage. As a result, the researcher responsible for randomization was consulted, and it was determined that the pregnant woman was assigned to the Placebo group.

In the Placebo group, the most prevalent complications were nausea (15.4%) and headache (11.5%). In the NAC group, headache (8.0%), drowsiness (12.0%), and a single report of dark urine (4.0%) were observed. Regarding serious adverse events, no maternal or fetal deaths were recorded in either arm of the study, although one case of spontaneous abortion (3.8%) occurred in the Placebo group. As for neonatal outcomes, the rates of admission to the neonatal intensive care unit (NICU) were 13.0% (*n* = 3) in the Placebo group and 16.7% (*n* = 4) in the NAC group (Table 5).

## 3. Discussion

In this study, we investigated an unadjusted, exploratory analysis that showed NAC supplementation to be associated with a longer gestational age at birth; however, this difference did not remain statistically significant after adjustment for relevant covariates (adjusted mean difference 0.92 weeks; 95% CI: −0.16 to 2.00; *p* = 0.093). These findings should therefore be interpreted with caution, given the exploratory nature of this pilot study, its limited statistical power, and the loss of significance after adjustment. If this trend were confirmed in adequately powered trials, a modest extension of gestational age could be hypothesized to carry neonatal relevance, for instance, by reducing the risk of extreme prematurity or allowing more time for antenatal corticosteroids to exert their full effect on fetal lung maturation [5], but this remains a hypothesis to be tested, not a demonstrated clinical benefit of the present pilot study.

In PE, the only definitive treatment is termination of pregnancy, which is indicated according to the severity of maternal disease and fetal conditions, as established by guidelines such as those of the American College of Obstetricians and Gynecologists and the World Health Organization [5,19]. Thus, the high frequency of surgical delivery in both groups suggests that the intervention does not alter the clinical determinants of delivery route, particularly in a setting where decision-making is primarily guided by maternal–fetal safety.

NAC-based therapeutic intervention has gained attention due to its role as a glutathione precursor, as well as its broader redox actions, functioning both as a direct scavenger of reactive oxygen species and as a regulator of intracellular redox homeostasis. A recent review highlights that these mechanisms, combining antioxidant and anti-inflammatory activity, position NAC as a promising non-hormonal therapeutic candidate in various chronic gynecologic conditions associated with immune dysregulation and inflammation [20].

The first trial using NAC was conducted in pregnant women with severe preeclampsia in the Netherlands with a sample of 38 women (19 in the Placebo group vs. 19 in the Intervention group), utilizing a short-term supplementation regimen (median of 6 days in the Intervention group and 5 days in the Placebo group). A higher dosage was used (1800 mg/day, divided into three 600 mg doses), and no effect on gestational age at birth was observed [21], in contrast to the findings presented here.

Another randomized clinical trial conducted with pregnant women with PE in Egypt involved an intervention group of 50 women who received 400 mg of NAC in addition to standard treatment—consisting of antihypertensive therapy (using methyldopa or calcium channel blockers in cases of uncontrolled hypertension) and magnesium sulfate in cases of eclampsia—and a control group of 50 pregnant women with PE who received only standard treatment. Contrary to our findings, Motawei et al. (2016) [22] observed that the supplemented group had offspring with statistically higher birth weights compared to the control group (2560 ± 590 g vs. 1980 ± 420 g, *p* < 0.001).

Comparing the three trials that investigated NAC in preeclampsia, important differences emerge across three dimensions that may explain the discrepancy in results: dose, disease severity at inclusion, and duration of supplementation. Regarding dose, the present study used 600 mg/day, an intermediate value between the lower dose employed by Motawei et al. (2016) [22] and the higher dose used by Roes et al. (2006) [21]. As for disease severity at inclusion, the Dutch trial exclusively included cases of already established severe preeclampsia requiring urgent clinical stabilization, a scenario in which the outcome of interest was short-term pregnancy maintenance. In contrast, the Egyptian trial and the present study included cases of preeclampsia at a less advanced stage, allowing for follow-up over a longer period.

This difference in severity is directly reflected in the duration of supplementation. In the Dutch trial, treatment lasted only 5–6 days (median), a timeframe consistent with the rapid progression of severe disease. In the present study, supplementation was maintained for a considerably longer period (median 74–78 days), whereas Motawei et al. (2016) [22] assessed outcomes after 4–6 weeks of treatment. These differences suggest that the timing of intervention may be a more relevant determinant of the response to NAC than dose alone. It is possible that prolonged supplementation, even at an intermediate dose, has greater potential to modulate oxidative stress processes over the course of the disease.

This hypothesis is consistent with the pharmacokinetic profile of NAC, characterized by low oral bioavailability (6–10%) and a short half-life (approximately 5.6 h) [23], which requires repeated and sustained exposure to promote relevant accumulation of glutathione precursors. Accordingly, brief treatment courses (5–6 days), even at a high dose, as in the Dutch trial, may be insufficient to generate a cumulative antioxidant effect, particularly in the context of already severe, established disease. Prolonged supplementation, as in the present study and the Egyptian trial, could instead favor a greater cumulative effect over time. This hypothesis, however, remains speculative and requires confirmation in trials that directly compare dose, timing of initiation, and treatment duration within the same population, ideally with sequential measurement of glutathione and other oxidative stress markers.

Low birth weight (LBW) is one of the most frequent neonatal complications in PE, generally resulting from uteroplacental insufficiency and the need for early termination of pregnancy, often the only effective measure to control the disease [24]. A systematic review with meta-analysis reported the prevalence of 39.7% of low birth weight among newborns born to mothers who developed PE [25].

A study conducted in Brazil reported that 32.7% of newborns born to mothers with PE had low birth weight (LBW) [26]. Furthermore, data from a large population-based prospective cohort study conducted in 2021, which included more than 200,000 Chinese women, demonstrated that PE and gestational hypertension were significantly associated with LBW, particularly when these conditions developed early in pregnancy [27]. The study also identified an increased risk of LBW and small for gestational age (SGA) births among the offspring of mothers with PE. The existing literature suggests that reduced uteroplacental blood perfusion and endothelial dysfunction—both consequences of elevated maternal blood pressure—contribute to an increased risk of fetal growth restriction [27,28,29].

In the present trial, newborn nutritional status and growth percentiles were assessed using the INTERGROWTH-21st standard. The low prevalence of fetal growth restriction and small-for-gestational-age infants observed in this trial is consistent with the predominantly late-onset phenotype of preeclampsia in our cohort, which is mechanistically distinct from early-onset preeclampsia and less frequently associated with severe placental insufficiency. Furthermore, it is worth noting that low birth weight is an important indicator of a child’s vulnerability to risks such as mortality and morbidity, including growth and developmental delay, metabolic syndrome, cardiovascular disorders, diabetes mellitus, and obesity. In addition, LBW contributes to higher hospital costs for families and the country [30].

It is important to note that in the trial conducted in the Netherlands [21], birth weights were considerably lower than those observed in the present study and lower than those reported by Motawei (2016) [22]. In that trial, birth weights were 1030 g (420–1800) in the Placebo group vs. 823 g (410–1830) in the Supplemented group, with no identified impact of NAC on this outcome, even at higher doses (1800 mg/day). These findings reinforce the hypothesis that NAC’s efficacy in PE may depend on disease severity.

Although no impact of NAC on the mode of delivery or birth weight was observed, the unadjusted difference in gestational age represents a secondary, exploratory finding that warrants further investigation, particularly since this association did not remain significant after adjustment for covariates in the present pilot study. Extending gestational age is a primary therapeutic goal in pre-eclampsia, as it is directly associated with reductions in neonatal morbidity and mortality. In the study by Roes et al. (2006) [21], even with a NAC dose approximately three times higher than that used in the present study, no prolongation of gestational age was observed. A possible explanation for the discrepancy is that the population evaluated in that study consisted of pregnant women with severe PE, which raises the hypothesis, not established by the present data, that any association between NAC and gestational age could be more pronounced in less advanced stages of the disease, where the redox imbalance is not yet as pronounced.

Another hypothesis that may explain the difference between the results is related to the pharmacokinetics of this compound. NAC exhibits rapid absorption after oral administration, with peak plasma levels in approximately 1–2 h, but with low bioavailability (< 10%) due to extensive first-pass hepatic metabolism and a relatively short half-life of around 5–6 h [17]. The supplements used in the two trials that also aimed to evaluate their effect in pregnancies with PE [21,22] were administered as effervescent tablets, whereas in the present study, they were administered as capsules.

From a pharmacokinetic point of view, effervescent tablets, because they are already dissolved at the time of ingestion, provide faster absorption, resulting in higher, but shorter-duration, plasma peaks. In contrast, capsules require intragastric disintegration and dissolution, which confer a slower, more gradual absorption profile [31].

Considering the pathophysiology of PE, characterized by chronic and continuous oxidative stress, maintaining more stable and sustained plasma levels of antioxidants may be more beneficial than high peaks followed by rapid drops, a more common pattern in effervescent formulations, especially due to the short half-life of NAC [17].

Assessing maternal outcomes, the impact of NAC on blood pressure control was not evidenced. It is known that elevated blood pressure is a classic clinical outcome of PE and, in fact, the fundamental pillar for establishing its syndromic diagnosis. In the present study, the absence of alteration in these parameters suggests that the action of NAC occurs through pathophysiological pathways distinct from those implicated in the genesis of maternal hypertension. Similarly, Roes et al. (2006) [21] did not find disease-stabilizing effects with the use of NAC in women with severe early-onset hypertension and/or HELLP (Hemolysis, Elevated Liver enzymes, Low Platelets). In contrast, Motawei and colleagues (2018) [32] observed that, after 4 and 6 weeks of intervention, the group treated with 400 mg of NAC had significantly lower systolic and diastolic blood pressure values than the control group. However, when evaluating blood pressure levels in the Placebo group at the end of the experiment, it was confirmed that this group also had a statistically significant decline when compared to baseline. It should be noted that the Placebo group remained subjected to standard treatment for PE (administration of 250 mg of methyldopa).

The results demonstrate that this antihypertensive drug is effective in controlling blood pressure in pregnant women with preeclampsia, being indicated as a standard intervention by official guidelines [5]. Its effectiveness in controlling blood pressure in pregnant women with PE and its safety for the fetus are widely documented, both in large-scale clinical trials [31] and in Cochrane systematic reviews [33], which corroborates the hemodynamic stability observed in our sample.

In the present study, the effect of NAC was also evaluated on several serum markers of oxidative stress, including enzymatic markers (superoxide dismutase (SOD), catalase (CAT), and myeloperoxidase (MPO)), lipid peroxidation (MDA), hydrogen peroxide (H_2_O_2_), and the endogenous antioxidant thiol tGSH in its oxidized and reduced forms, as well as the GSH/GSSG ratio. However, no significant differences were observed between the groups evaluated. These findings suggest that NAC supplementation at the dosage used in this study may not have been sufficient to elicit detectable changes in systemic biomarkers of oxidative stress. Furthermore, the pharmacokinetics of orally administered NAC should be considered, as this molecule has relatively low bioavailability due to extensive first-pass hepatic metabolism. Furthermore, physiological changes inherent to pregnancy, such as increased plasma volume and modifications in the metabolism and clearance of exogenous compounds, can influence the absorption, distribution, and systemic availability of NAC, potentially limiting its measurable effects on circulating biomarkers.

In contrast, Motawei et al. (2016) [22] reported a significant increase in serum SOD and glutathione peroxidase (GPx) levels following NAC supplementation. However, it is important to note that these authors used a group of healthy pregnant women as a reference, which may have exaggerated the observed differences in enzyme levels [21].

Specifically in the present study, NAC supplementation was expected to promote an increase in serum GSH levels in supplemented pregnant women, considering that a previous survey conducted by our group demonstrated a positive association between placental GSH levels and better perinatal outcomes, including birth weight, head circumference, chest circumference, and gestational age at birth [12]. However, it is important to emphasize that these results were obtained from placental tissue, suggesting that direct evaluation of this tissue may be a more sensitive approach for detecting changes in the local redox state. In this context, the analysis of biomarkers in placental tissue may be particularly relevant in clinical trials investigating antioxidant interventions in pregnancies complicated by PE.

It should also be noted that the absence of significant changes in serum markers of oxidative stress contrasts with the unadjusted difference in gestational age observed between groups, a difference that, as noted above, did not persist after adjustment for covariates. Although this finding may suggest localized effects of NAC, particularly in the placental microenvironment, this remains a speculative hypothesis and should be interpreted with caution. Experimental evidence indicates that NAC may exhibit affinity for trophoblastic tissue [34], raising the possibility that its effects occur predominantly at the tissue level and are not readily reflected in circulating biomarkers.

Additionally, the present study evaluated the effect of NAC on serum inflammatory biomarkers, including pro-inflammatory (IL-17, TNF-α, and IL-22) and anti-inflammatory (IL-10) cytokines, which have been frequently described in studies on PE [35,36,37,38]. However, no significant alterations in these mediators were observed. Although experimental studies indicate that NAC may modulate the NF-κB signaling pathway by regulating the intracellular redox state, the findings of the present study do not allow confirmation of this mechanism at the systemic level. Thus, future investigations could explore additional molecular markers, such as TNF receptor expression and components of the NF-κB pathway at the tissue level, to elucidate the potential effects of NAC in specific cellular environments and deepen understanding of its therapeutic potential in PE [39].

Finally, in this research, the role of NAC in specific clinical symptoms of PE was also evaluated, including headache, scotomas, and epigastric pain, with no improvement in these parameters when comparing the groups of pregnant women supplemented with NAC or placebo. In the trial by Roes et al. [21], the manifestation of adverse effects (nausea, vomiting, and gastric discomfort) was also observed, but the authors emphasize that the adverse effects reported are likely more related to the disease itself or to the effervescent characteristics of the tablets than to NAC itself.

It is important to emphasize several strengths of the present study: it is a pilot trial that evaluated a wide range of variables, some not yet described in other studies, and included parameters related to the mother–child dyad. Furthermore, when compared to other trials involving NAC in pregnancies complicated by PE, this study stands out for maintaining the intervention for a longer average duration.

However, this study has limitations, including the evaluation of NAC’s action in only one biological matrix (blood), variable supplementation duration, and the lack of measurement of biochemical markers associated with worsening PE.

Furthermore, although the initial sample size calculation anticipated the inclusion of 120 participants, recruitment was concluded with 51 women due to logistical challenges and highly specific eligibility criteria, established to maintain strict methodological rigor. The lower-than-estimated number of participants, combined with the assessment of multiple secondary outcomes and biomarkers—factors that increase the risk of type I error due to multiple comparisons—limits the statistical power of the study and warrants caution in interpreting the exploratory findings, particularly those related to gestational age at birth.

Another relevant point is that, although an intention-to-treat analysis was initially planned, it could not be performed due to the absence of follow-up information, including data related to delivery and neonatal outcomes, among participants who transferred their prenatal care to other health services. As inputting these missing data would require assumptions not supported by the available information, a per-protocol analysis was chosen instead; however, this approach may affect the generalizability of the results. Additionally, the duration of NAC supplementation varied among participants, reflecting the clinical reality of recruitment at different gestational periods and the timing of delivery. This variability may have influenced treatment exposure and potentially contributed to differences in biological response.

It should be emphasized that adherence was assessed by self-report, a method subject to recall and social desirability biases, which precludes objective confirmation of treatment adherence. Therefore, future studies should incorporate objective adherence monitoring strategies, standardization of treatment duration, and intention-to-treat analyses with adequate follow-up, in order to strengthen the evidence on NAC supplementation in preeclampsia.

To further advance this area of research, it is suggested that the expression of receptors, such as TNF receptors, and cellular mediators, such as the nuclear factor NF-κB, be investigated to identify potential therapeutic strategies to inhibit these mechanisms. Additionally, the need for multicenter clinical trials with larger sample sizes to explore higher NAC doses and to consolidate its role in the management of PE is highlighted.

We emphasize that, although the trial was registered retrospectively, which may raise concerns regarding the risk of bias, all study procedures, including the protocol, primary and secondary outcomes, eligibility criteria, intervention, and statistical analysis plan, were predefined and approved by the Research Ethics Committee prior to the initiation of recruitment and were followed as planned throughout the study. Furthermore, the expansion of the inclusion criterion from the 24th to the 22nd week of gestation was approved by the Ethics Committee prior to its implementation and did not entail any modifications to the previously established outcomes, intervention, or analyses.

## 4. Materials and Methods

### 4.1. Ethical Aspects

There was no financial compensation for participation in this study. This investigation follows the guidelines of the Consolidated Standards of Reporting Trials (CONSORT) [40]. The study protocol was approved by the Research Ethics Committee (REC) of the Federal University of Alagoas, under registration number 4,257,473, in accordance with the ethical principles of the Declaration of Helsinki of 1964 and its subsequent amendments.

All participants were informed about the study procedures and signed the Informed Consent Form (ICF) before inclusion. The clinical trial was retrospectively registered in the Brazilian Clinical Trials Registry (ReBEC) (https://ensaiosclinicos.gov.br/, Registration date: 6 November 2024), under the identifier RBR-6n5p6gw. Registration occurred after the start of participant recruitment due to administrative and operational delays. All study procedures, including outcomes and protocol, were defined a priori and approved by the Research Ethics Committee before the start of the study. Although the initial protocol approved by the Research Ethics Committee (REC) stipulated inclusion from the 24th week of gestation, the criteria were expanded to the 22nd week during the recruitment phase. This adjustment was implemented to encompass cases of early-onset pre-eclampsia treated at the referral center, while maintaining consistency across all intervention procedures.

It should be noted that neither the retrospective registration of the trial nor the expansion of the inclusion criterion to pregnant women from the 22nd week of gestation onward compromised the methodological conduct of the study, since the protocol, outcomes, eligibility criteria, intervention procedures, and statistical analysis plan were predefined and approved by the Research Ethics Committee prior to the initiation of participant enrollment, and were consistently followed throughout the study.

### 4.2. Study Design

This is a randomized, double-blind, placebo-controlled pilot study conducted from 2 August 2021 to 25 August 2023.

### 4.3. Sample Size Calculation

The sample size was calculated using G*Power software, version 3.1.9.2. Based on an expected 50% reduction in the cesarean delivery rate, adopted as the primary outcome, between the exposed and unexposed groups, and considering a statistical power of 80% with a 95% confidence level, a total of 120 pregnant women with pre-eclampsia were determined. Participants were randomly assigned to two arms: 60 in the intervention group (NAC supplementation) and 60 in the control group (placebo).

### 4.4. Participants

The study was conducted at Professor Alberto Antunes University Hospital (AAUH), a tertiary referral center for high-risk pregnancies in Alagoas, located in Maceio, Brazil. For convenience, sampling was performed using a non-probabilistic approach. Participants were recruited through direct invitations to women involved in hospital activities or attending prenatal screening. It is noteworthy that collection teams were available during all scheduled prenatal care hours, and all eligible pregnant women were invited to participate. All pregnant women presenting with alterations in blood pressure levels verified during screening or who already had a diagnosis of preeclampsia (PE) signaled in their medical records were identified and invited to participate. Following the initial invitation, the pregnant women underwent a detailed evaluation to confirm the inclusion criteria, including a urine dipstick test to verify proteinuria in those whose medical records did not include this information.

This study included pregnant women between 22 and 32 weeks of gestation who had a confirmed diagnosis of pre-eclampsia (PE) and were receiving prenatal care at AAUH. Exclusion criteria included multiple pregnancies, maternal neurological disorders or psychiatric disorders, and comorbidities with the potential to affect gestational outcomes (diabetes mellitus, cardiovascular diseases, urinary tract infection, kidney disease, autoimmune disorders, liver disease, sexually transmitted infections). Women who reported alcohol and/or other drug use, smoking, or the use of supplements with potential antioxidant and/or anti-inflammatory effects (such as vitamin C, vitamin E, selenium, resveratrol, beta-carotene, among others) were also excluded, with the exception of supplements whose use is already recommended during pregnancy (ferrous sulfate/elemental iron, folic acid/methyl folate, and omega-3). Additionally, pregnancies with fetuses diagnosed with major congenital malformations (cardiac, renal, or hepatic) or genetic syndromes prior to recruitment were excluded. In cases where such conditions were diagnosed during the trial, participants were withdrawn from the study.

Chronic hypertension and obesity were not adopted as exclusion criteria. This decision was made to ensure that the study population remained representative of the high-risk demographic profile served at our referral center. Furthermore, excluding these prevalent comorbidities would excessively restrict the sample size and limit the external validity of the clinical findings.

Preeclampsia was defined according to the International Society for the Study of Hypertension in Pregnancy criteria (ISSHP) [41]. For pregnant women included without a prior diagnosis, proteinuria was assessed using a reagent strip test on a urine sample. In cases where the diagnosis was already on record, it was confirmed by the institution’s clinical team based on hypertension associated with proteinuria or, in the absence thereof, by the presence of organ dysfunction, such as thrombocytopenia (<100,000/mm), elevated liver enzymes, renal dysfunction, pulmonary edema or neurological symptoms (persistent headache, visual disturbances and seizures) [41].

Although the ISSHP guidelines recommend, whenever possible, the quantification of proteinuria through the urinary protein/creatinine ratio or 24 h urine collection, the reagent strip test (qualitative method) was chosen due to the limited availability of laboratory resources for quantitative testing at the time of screening within the service’s routine care, as well as the practicality of this method. It should be emphasized that this is a widely used method in clinical practice for initial screening and is accepted by ISSHP itself as a screening tool when quantitative methods are not readily available. It is acknowledged, however, that the qualitative nature of the method constitutes a limitation regarding the precision of proteinuria quantification.

### 4.5. Randomization

Participants were randomized to the NAC (intervention) or placebo group using a permuted block randomization scheme. The randomization sequence was generated in blocks of sizes 4–8, using StatsDirect software, version 3.0 (StatsDirect Ltd., Merseyside, UK). This generated sequence was strictly managed by an independent researcher, not involved in data collection or clinical evaluation, who prepared sequentially numbered, opaque, sealed envelopes containing the allocation group. All researchers involved in the screening or clinical trial remained blinded to the randomization method and could not predict the group to which any participant would be allocated.

### 4.6. Intervention

Once recruited, supplementation was carried out from the time of inclusion until delivery. At each visit, pregnant women received sealed, opaque containers containing either 600 mg NAC capsules (NAC group) or capsules containing 2% colloidal silicon dioxide and 98% microcrystalline cellulose (placebo group). The capsules for both groups were identical in size, shape, color, and odor, and were packaged in identical containers. The formulas were compounded and encapsulated by a commercial compounding pharmacy in accordance with Good Manufacturing Practices, ensuring that the research team remained blind to the intervention. All pregnant women were instructed to take one capsule daily in the morning [42].

The selection of the 600 mg dose of *N*-acetylcysteine was based on its well-established safety profile and human pharmacokinetic data, which demonstrate predictable absorption, limited accumulation, and favorable tolerability. Although pharmacokinetic data in pregnant women remain scarce, available studies indicate increased maternal clearance and significant placental transfer, supporting the biological plausibility of the intervention and highlighting the need for further dose-optimization studies in this population. In addition, the present study aimed to delimit its action as a dietary supplement. Therefore, the maximum dose allowed by Brazilian legislation for this substance in this category was adopted. In Brazil, doses of NAC greater than 600 mg are classified exclusively as a medicine and require specific health registration and a medical prescription.

Throughout the trial, the pregnant women continued the same supplementation regimen established at the time of their inclusion in the study. The research team monitored adherence during routine prenatal consultations, held monthly or biweekly, with the interval between consultations determined by the obstetrician. At each encounter, adverse events were recorded, including headache, vomiting, nausea, diarrhea, and other clinical complications. To promote adherence, informative messages were sent daily to remind the pregnant women to take their capsules. Along with the capsules, they received a booklet with a specific section for daily intake recording. Additionally, they were instructed to bring the containers to each visit to allow for adherence assessment. The participants received 1 or 2 bottles of 30 capsules each, depending on the interval between prenatal consultations.

### 4.7. Outcome

Sociodemographic data and personal and family history were recorded. Anthropometric measurements (height, weight, and body mass index [BMI]), systolic and diastolic blood pressure, and proteinuria were assessed from a single urine sample collected before the interview. Weight was measured with an in-Body^®^ (InBody Co., Ltd., Seoul, Republic of Korea) digital scale, and height with a Cescorf^®^ (Cescorf Equipamentos para Esporte Ltda., Porto Alegre, RS, Brazil) portable stadiometer. These variables were used to calculate the participants’ BMI; the anthropometric diagnosis was based on the method of Atalah et al. [43]. In addition, pre-gestational weight (used to calculate pre-gestational BMI) and weight gain during pregnancy were assessed in accordance with the recommendations of the Institute of Medicine (IOM-USA) [44]. Gestational weeks at the time of admission to the trial were also recorded; this was determined based on the Last Menstrual Period (LMP) or, when unavailable or uncertain, extracted from obstetric records based on early ultrasound findings. Concomitant treatments and co-interventions were also tracked during assessments, especially antihypertensives, magnesium sulfate, corticosteroids, and hospitalization status.

The participants’ blood pressure was measured three times at 10 min intervals, and the average was recorded. Urine protein levels were assessed using the Sensi 10 urine reagent strips (URIT Medical Electronic Co., Ltd., Guilin, China), according to the manufacturer’s recommended procedures. Proteinuria was defined as a reading of at least 1^+^ on the dipstick test.

Blood samples were collected at two time points (baseline and at the end of the clinical trial) by qualified professionals (hospital staff) and processed in accordance with biosafety standards to minimize unnecessary discomfort for the study participants. The material was collected in Vacutainer^®^ tubes (Becton, Dickinson and Company, Franklin Lakes, NJ, USA), according to standard human blood collection protocols. Then, the blood samples for biochemical assays (redox imbalance and cytokine levels) were kept on ice in a thermal box until centrifugation (4000 rpm for 10 min at 4 °C). Subsequently, the samples were aliquoted for analysis and stored at −80 °C.

Data regarding childbirth and the newborn were collected from physical and/or electronic medical records. These included birth weight (g), length (cm), head circumference (cm), chest circumference (cm), and sex. Additionally, the mode of delivery, Apgar scores at one and five minutes, and postpartum complications were recorded.

The nutritional status of newborns was determined using percentiles established by the INTERGROWTH-21st standard [45]. Weight-for-age, height-for-age, and head circumference-for-age values were calculated. The ratio between head circumference (HC) and chest circumference (CC), an index of nutritional status, was also calculated. Normal values were defined as CC/HC ratios equal to or greater than 1 [46].

Gestational age (GA) was classified according to the World Health Organization criteria [47]: preterm newborns (GA < 37 weeks), term newborns (GA 37–42 weeks), and post-term newborns (GA > 42 weeks). The Apgar score (appearance, pulse, grimace, activity, respiration) was adopted because it can assess the newborn’s health status at birth, with a score of less than 7 at both minutes (first and fifth) considered at risk [48].

The following redox imbalance biomarkers were analyzed (in duplicate): superoxide dismutase (SOD), using the SOD Assay kit (Nunc^®^, Roskilde, Denmark); hydrogen peroxide (H_2_O_2_), according to Pick and Keisari (1980) [49]; catalase (CAT), following the colorimetric method, previously described by Aebi (1984) [50]; total glutathione (tGSH) and oxidized glutathione (GSSG) were determined according to the Colorimetric Detection Kit (Invitrogen™). The ratio between them (GSH/GSSG) was calculated, and reduced glutathione (GSH) was determined by the following formula: GSH = tGSH − (2 × GSSG). All these biomarkers were identified by spectrofluorometry, in duplicate, with wavelengths specified for each method. Malondialdehyde (MDA) levels were measured by reversed-phase high-performance liquid chromatography (HPLC) with ion-pairing and ultraviolet detection at 270 nm and expressed in ng/µL [51].

The following levels of interleukin-10 (IL-10), IL-17, IL-22, and tumor necrosis factor alpha (TNF-α) were measured using the enzyme-linked immunosorbent assay (ELISA) with the PeproTech^®^ kit (PeproTech Brasil FUNPEC, Ribeirão Preto, São Paulo, Brazil), according to the manufacturer’s instructions. Absorbance was read at 450 nm in an ELISA plate reader, and results were expressed in pg/µL. Myeloperoxidase (MPO) activity was measured by adapting the method previously described by Bradley et al. (1982) [52], and results were expressed as U/µL. One MPO unit was defined as the amount of enzyme required to decompose 1 μmol of H_2_O_2_ [52].

The primary outcome was the perinatal outcome of delivery method (cesarean section), as defined a priori in the original protocol and used to calculate sample size. Secondary outcomes included markers of oxidative stress and inflammation, as well as maternal outcomes (blood pressure levels and symptoms of preeclampsia (scotomas, epigastric pain, headache), as well as progression to eclampsia or other maternal complications at the time of delivery) and other perinatal outcomes: gestational age at birth, birth weight, weight for gestational age, length for gestational age, head circumference and the head Chest circumference/head circumference ratio (CC/HC), and Apgar score.

Additionally, at each subsequent visit, potential adverse effects were assessed by screening for the following symptoms: headache, vomiting, nausea, diarrhea, or any symptoms reported by the pregnant woman that arose after starting to use the capsules.

### 4.8. Statistical Analysis

Data analysis was performed following a per-protocol approach, including only participants who completed the entire supplementation regimen and clinical follow-up until delivery. Data were processed using SPSS (Statistical Package for the Social Sciences) version 20.0. The normality of the distribution was assessed using the Lilliefors test, and the homogeneity of variances was assessed using Levene’s test. Results for continuous variables were presented as mean ± standard deviation. The chi-square test was used to analyze the primary outcome. Redox imbalance and inflammation markers were assessed using mixed ANOVA. Student’s *t*-test was used to examine maternal and fetal outcomes. In all tests, a significance level of 5% was adopted.

A longitudinal analysis model with Generalized Estimating Equation (GEE) was used to estimate the effect of NAC supplementation on typical preeclampsia symptoms, namely, elevated blood pressure, scotomas, headache, and epigastric pain, using repeated measures corresponding to the number of follow-up visits for each pregnant woman. A crude regression model was used to estimate prevalence ratios with 95% confidence intervals (95% CI), with statistical significance defined as *p* < 0.05.

For variables that showed statistical significance (*p* < 0.05), the magnitude of the effect (effect size) of the intervention was evaluated. Cohen’s d was used, calculated from the combined means and standard deviations (pooled standard deviation), according to Cohen’s criteria (1988), where values of 0.2, 0.5, and 0.8 represent small, moderate, and large effects, respectively.

In addition, to evaluate the effect of NAC supplementation on variables with *p* < 0.05, controlling for possible confounding variables, an Analysis of Covariance (ANCOVA) was performed. The model included maternal age, body mass index (pre-gestational and current BMI), and systolic and diastolic blood pressure as covariates. The assumptions of linearity and homogeneity of variances were verified before running the model.

## 5. Conclusions

In conclusion, NAC supplementation did not result in a statistically significant difference in mode of delivery (primary outcome) between the NAC and placebo groups, the main finding of this study. Likewise, no impact of NAC was observed on maternal blood pressure control or on classic PE symptoms. As a secondary exploratory finding, a possible trend toward prolonged pregnancy was observed in the NAC group; however, this result should be interpreted with caution, given the neutrality of the primary outcome and the exploratory nature of the analysis.

## Figures and Tables

**Table 1:** Characterization of pregnant women with preeclampsia (PE) treated at a university hospital in the state of Alagoas, Brazil, from 2022 to 2023.

Variables	Placebo Group N = 26 (%)	NAC Group N = 25 (%)	*p*-Value
**Age (years)**			
≤19	1 (3.85)	3 (12.00)	0.523
20–34	17 (65.38)	16 (64.00)	
≥35	8 (30.77)	6 (24.00)	
**Region of residence**			
Capital	13 (50.00)	14 (56.00)	0.781
Other cities	13 (50.00)	11 (44.00)	
**Number of members in the household**			
≤5	6 (23.08)	6 (24.00)	0.990
>5	20 (76.92)	19 (76.00)	
**Monthly family income** **(R$)**			
≤1 minimum wage	16 (61.54)	13 (54.17)	0.775
>1 minimum wage	10 (38.46)	11 (45.83)	
No information		1	
**Hypertension prior to pregnancy**			
Yes	5 (19.23)	6 (24.00)	0.733
No	21 (80.77)	19(76.00)	
**Works outside home**			
Yes	10 (38.46)	7 (28.00)	0.555
No	16 (61.54)	18 (72.00)	
**Education level**			
Incomplete elementary education	5 (19.23)	7 (28.00)	0.242
Complete elementary education	1 (3.85)	5 (20.00)	
Incomplete high school	1 (3.85)	2 (8.00)	
Complete high school	16 (61.54)	9 (36.00)	
Incomplete/complete higher education	3 (11.54)	2 (8.00)	
**Ethnicity**			
White	4 (15.38)	1 (4.00)	0.336
Mixed race	18 (69.23)	22 (88.00)	
Black	3 (11.54)	2 (8.00)	
Others	1 (3.85)	0 (0)	
**Stable union**			
Yes	18 (69.23)	17 (68.00)	1.00
No	8 (30.77)	8 (32.00)	
**Start of prenatal care**			
First trimester	19 (76.00)	18 (72.00)	1.000
Second/third trimester	6 (24.00)	7 (28.00)	
No information	1		
Methyldopa use *			
Yes	21 (95.45)	21 (95.45)	1.00
No	1 (4.55)	1 (4.55)	
No information	4	3	
**Use of ferrous sulfate and folic acid**			
Yes	20 (76.92)	16 (66.67)	0.533
No	6 (23.08)	8 (33.33)	
No information		1	
**Classification of pre-pregnancy nutritional status** **(BMI-kg/m^2^)**			
Underweight	1 (4.00)	0 (0)	1.000
Normal weight	1 (4.00)	1 (4.20)	1.000
Overweight	6 (24.00)	5 (20.80)	1.000
Obesity	17 (68.00)	18 (75.00)	0.754
Missing pre-gestational data	1	1	
**Classification of gestational nutritional status (BMI-kg/m^2^)**			
Underweight	1 (3.85)	1 (4.00)	1.00
Normal weight	3 (11.54)	3 (12.00)	0.998
Overweight	5 (19.23)	4 (16.00)	0.955
Obesity	17 (65.38)	17 (68.00)	0.977

A Pearson’s chi-square test, *p* < 0.05, is significant. BMI: body mass index. * represents methyldopa use ranged from 250 mg, two to three times daily, according to Brazilian medical guidance.

**Table 2:** Assessment of the effect of *N*-acetylcysteine (NAC) supplementation on fetal and maternal outcomes of pregnancies with preeclampsia (PE) treated at a university hospital in the state of Alagoas, Brazil, from 2022 to 2023.

		Placebo Group			NAC Group		*p*-Value
	N	Mean	SD	N	Mean	SD	
Gestational age at birth (weeks)	23	37.39	1.96	24	38.45	1.51	0.044
Birth weight (grams) ^a^	23	3100.09	656.92	24	3105.89	510.40	0.97
Weight percentile for gestational age (Intergrowth-21)	23	62.73	27.78	24	54.29	26.70	0.29
Height percentile for gestational age (Intergrowth-21)	23	50.34	28.37	24	54.68	30.86	0.61
Head circumference percentile for age (Intergrowth-21)	20	78.76	26.67	22	77.33	23.80	0.85
Chest circumference/head circumference ratio	15	0.93	0.071	17	0.94	0.046	0.13
Apgar at the 1st minute	21	7.81	1.32	24	8.12	0.74	0.32
Apgar at the 5th minute	21	8.91	0.54	24	9.17	0.56	0.12
Initial systolic blood pressure	23	136.92	16.02	24	139.98	21.92	0.20
Early diastolic blood pressure	23	83.23	10.84	24	84.39	16.85	0.77
Systolic blood pressure on the 1st follow-up	23	126.13	12.93	24	124.87	15.90	0.18
Diastolic blood pressure on the 1st follow-up	23	73.87	15.9	24	77.53	9.47	0.47
Systolic blood pressure at last visit	23	140.80	18.15	24	133.50	16.29	0.60
Diastolic blood pressure at last visit	23	87.73	7.67	24	83	10.14	0.55
Mode of delivery *	***n***	**%**		***n***	**%**		
Vaginal	8	34.78	-	5	20.83	-	0.23
Cesarean	15	65.22	-	19	79.17	-	
Preterm birth							
Yes	8	34.78		5	20.83	-	0.34
No	15	65.22		19	19.17	-	

Student’s *t*-test; * Pearson chi-square; ^a^ An analysis of Covariance (ANCOVA) was performed, adjusted for gestational age at admission, duration of supplementation, maternal age, BMI, and blood pressure levels at recruitment. The full model was statistically significant (F(8,28) = 2.624; *p* = 0.028; *η*^2^p = 0.429). However, treatment allocation (NAC vs. control) was not independently associated with gestational age at birth after adjustment for covariates (F(1,28) = 0.144; *p* = 0.707; *η*^2^p = 0.005). The adjusted mean difference between groups was 0.92 weeks (95% CI: −0.16 to 2.00).

**Table 3:** Evaluation of the effect of *N*-acetylcysteine (NAC) supplementation on stress and inflammation biomarkers in pregnant women with preeclampsia (PE) treated at a university hospital in the state of Alagoas, Brazil, from 2022 to 2023.

	Before			After		
Oxidative Stress or Inflammation Markers	*n*	Mean	SD	Mean	SD	*p*-Value
**Malondialdehyde (MDA) (ng/µL)**						
Placebo	24	19.70	10.22	17.29	8.35	0.360
NAC	24	18.64	6.10	18.93	4.98	
**Superoxide dismutase (SOD) (U/µL)**						
Placebo	24	5844.38	3270.25	4563.66	2817.17	0.634
NAC	24	6522.09	2605.10	4737.34	3133.68	
**Catalase (CAT) (U/min)**						
Placebo	24	10.71	3.30	10.83	3.49	0.593
NAC	24	12.07	2.77	11.46	3.91	
**Hydrogen peroxide (H_2_O_2_) (nmol/mL)**						
Placebo	24	237.69	219.81	226.26	182.53	0.738
NAC	24	195.93	197.79	204.36	192.19	
**Reduced glutathione (GSH)**						
Placebo	20	123.30	31.44	117.32	24.38	0.212
NAC	17	122.34	41.33	135.02	28.14	
**Oxidized glutathione (GSSG)**						
Placebo	20	16.90	14.51	9.31	4.88	0.210
NAC	17	22.19	19.80	7.47	3.79	
**GSH/GSSG**						
Placebo	20	15.04	18.44	22.47	28.24	0.707
NAC	17	21.09	39.85	23.70	13.41	
**Myeloperoxidase (MPO) (U/µL)**						
Placebo	19	12.09	3.92	7.84	1.91	0.875
NAC	21	13.16	2.98	8.72	2.22	
**Tumor necrosis factor alpha (TNF-α) (pg/µL)**						
Placebo	23	1.40	0.52	2.16	1.77	0.509
NAC	21	1.21	0.62	1.69	0.92	
**IL-22 (pg/µL)**						
Placebo	23	0.78	0.37	1.19	0.79	0.604
NAC	20	0.79	0.30	1.05	0.69	
**IL-17 (pg/µL)**						
Placebo	24	9.96	2.88	7.38	2.47	0.348
NAC	21	11.79	3.22	8.24	2.63	
**IL-10 (pg/µL)**						
Placebo	24	15.90	7.37	10.09	5.06	0.239
NAC	21	20.47	5.49	12.54	4.04	

Mixed ANOVA; IL—interleukin.

**Table 4:** Crude analysis of the association between NAC supplementation and incidence of typical preeclampsia symptoms among pregnant women with PE treated at a university hospital in the state of Alagoas, Brazil, from 2022 to 2023.

Variable	Prevalence Ratio (PR)	Confidence Interval (CI)	*p*-Value
**Elevated pressure**	0.901	0.66–1.23	0.51
**Headache**	0.84	0.57–1.24	0.38
**Scotomas**	0.75	0.41–1.34	0.52
**Epigastric pain**	0.52	0.27–1.002	0.051

Note: Prevalence ratios were estimated using Generalized Estimating Equation (GEE).

**Table 5:** Analysis of safety and incidence of maternal and perinatal adverse events among pregnant women with PE supplemented with NAC or Placebo at a university hospital in Alagoas, Brazil, from 2022 to 2023.

Adverse Event	Placebo Group (*n* = 26)	NAC Group (*n* = 25)	*p*-Value *	Related To NAC?
**Maternal Mild Symptoms**				
Nausea	4 (15.38%)	0 (0.00%)	0.110	No
Headache	3 (11.54%)	2 (8.00%)	0.999	No
Drowsiness	0 (0.00%)	3 (12.00%)	0.108	Unlikely
Hyperpigmented urine	0 (0.00%)	1 (4.00%)	0.490	Possible **
**Serious Adverse Events (SAEs)**				
Miscarriage	1 (3.85%)	0 (0.00%)	0.999	No
Maternal Death	0 (0.00%)	0 (0.00%)	-	-
Neonatal Complications	(*n* = 23)	(*n* = 24)		
NICU Admission	3 (13.04%)	4 (16.67%)	0.698	No
Perinatal Death	0 (0.00%)	0 (0.00%)	-	-

* Fisher’s Exact Test was used due to small frequencies. ** Hyperpigmented urine is a known harmless metabolic excretion of NAC. The miscarriage occurred in the Placebo group, as confirmed by the randomization coordinator, and was deemed unrelated to any intervention.

## Data Availability

The original contributions presented in the study are included in the article, further inquiries can be directed to the corresponding authors. The datasets generated and analyzed during the current study are publicly available in the Zenodo repository: Silva, D.A.V. (2025). Impact of N-acetylcysteine (NAC) supplementation on serum biomarkers and perinatal outcomes in pregnancies with pre-eclampsia [Data set]. Zenodo. https://doi.org/10.5281/zenodo.18096095, accessed on 30 December 2025.

## Abbreviations

The following abbreviations are used in this manuscript:

AAUH	Alberto Antunes University Hospital
ANCOVA	Analysis of Covariance
ANOVA	Analysis of Variance
BMI	Body Mass Index
CAT	Catalase
CC	Chest Circumference
CC/HC	Chest Circumference/Head Circumference ratio
CI	Confidence Interval
CONSORT	Consolidated Standards of Reporting Trials
ELISA	Enzyme-Linked Immunosorbent Assay
GA	Gestational Age
GEE	Generalized Estimating Equation
GPx	Glutathione Peroxidase
GSH	Reduced Glutathione
GSSG	Oxidized Glutathione
HC	Head Circumference
HELLP	Hemolysis, Elevated Liver enzymes, Low Platelets
H_2_O_2_	Hydrogen peroxide
HPLC	High-performance liquid chromatography
ICF	Informed consent form
IL-10	Interleukin-10
IL-17	Interleukin-17
IL-22	Interleukin-22
IOM	Institute of Medicine
ISSHP	International Society for the Study of Hypertension in Pregnancy
IUGR	Intrauterine growth restriction
LBW	Low birth weight
LMP	Last menstrual period
MDA	Malondialdehyde
MPO	Myeloperoxidase
NAC	N-acetylcysteine
NF-κB	Nuclear Factor kappa B
NICU	Neonatal intensive care unit
PE	Preeclampsia
PR	Prevalence Ratio
REC	Research Ethics Committee
ReBEC	Registro Brasileiro de Ensaios Clínicos (Brazilian Clinical Trials Registry)
RONs	Reactive Oxygen and Nitrogen Species
SAEs	Serious adverse events
SD	Standard deviation
SGA	Small for gestational age
SOD	Superoxide dismutase
SPSS	Statistical Package for the Social Sciences
tGSH	Total glutathione
TNF-α	Tumor necrosis factor alpha
UFAL	Federal University of Alagoas
WHO	World Health Organization
